# Effect of Heat Treatment on the Microstructural Heterogeneity and Abrasive Wear Behavior of ASTM A128 Grade C Steel

**DOI:** 10.3390/ma17122884

**Published:** 2024-06-13

**Authors:** Oscar-Fabián Higuera-Cobos, María-Mercedes Cely-Bautista, Jairo-Alberto Muñoz-Bolaños

**Affiliations:** 1 Research Group CONFORMAT, Mechanical Engineering Program, Faculty of Engineering, Universidad del Atlántico, Puerto Colombia 081007, Atlántico, Colombia; mariacely@mail.uniatlantico.edu.co; 2 Department of Materials Science and Engineering EEBE, Universidad Politécnica de Catalunya, c/Eduard Maristany 10-14, 08019 Barcelona, Spain; jairo.alberto.munoz@upc.edu

**Keywords:** ASTM A128 steel, austenitic manganese steel, martensite, abrasive wear, carbides, decarburization

## Abstract

Microstructural heterogeneities of an ASTM A128 grade C steel subjected to heat treatments and their effect on abrasive wear behavior were investigated. The heat-treatment process involved different austenization times at 1050 °C and quenching media. To characterize the effects of heat treatment on the material’s microstructure and mechanical behavior, two microscopy techniques were used: optical microscopy (OM), and scanning electron microscopy (SEM). The chemical composition of the material was obtained using X-ray fluorescence (XRF) optical emission spectrometry. The variation in carbide composition was evaluated using X-ray Energy Dispersive Spectroscopy (EDS). To characterize the mechanical behavior of the steel, hardness measurements and abrasive wear tests were performed after homogenization annealing and quenching treatments. The results showed that the heat-treated samples developed a heterogeneous microstructure, with the presence of austenitic grains and Martensite around the surface of the samples induced by decarburization in both the protected and unprotected specimens. The specimens with lower decarburization presented less formation of Martensite and precipitated carbides, resulting in lower hardness values and higher abrasion resistance (solution treatment at 1080 °C for 1 h + sand protection + brine quenching).

## 1. Introduction

Austenitic Manganese steel, usually called “Hadfield steel”, was developed by Robert Hadfield in 1882 [1,2]. This material contains 1.2% of C and 12% of Mn. These steels have good properties related to their work-hardening capacity and high resistance to wear under high-stress conditions, toughness, and good workability, which makes them into good candidates for the industry, especially in the railway industry, steel mills, mining, and construction sectors, among others. However, one of the problems presented by machines such as impact crushers, hammer crushers, excavators, plowing equipment, and railroad tracks, among others, is their low resistance to abrasive wear, a problem that reduces the useful life of this equipment [1,2].

Abrasion resistance in Hadfield steels is not directly related to hardness, since it depends largely on their composition and the microstructure obtained after quenching [3,4]. These steels are very susceptible to decarburization processes, which enables an undesirable solid-state transformation to the detriment of its mechanical performance. The properties of Hadfield steels have been improved by the addition of TiC-reinforcing particles, or carbide-forming elements such as chromium and vanadium, that improve wear resistance, yet could lead to an increase in the embrittlement of the alloys, which is not desirable for some mechanical applications. In some cases, the addition of aluminum has been implemented to decrease the formation of carbides at grain boundaries, and thus increase the wear resistance at low stresses [5]. According to Tecza et al. [6], the presence of carbides in this type of alloy reduces the ductility of Hadfield steels due to the effect that carbides generate on grain boundaries, reducing their impact resistance. However, the segregation of acicular carbides especially rich in chromium and molybdenum, or by the formation of core–shell type structures, improves the resistance to abrasive wear [7].

Another approach that has been tried in this type of steel is the application of heat treatments to increase the apparent hardness, maintaining the toughness and machinability by minimizing the appearance of carbides at the grain boundaries by properly controlling the austenitization temperature, furnace environment, and cooling medium [8,9]. According to Bańkowski et al. [10] and Torabi et al. [11], the increase in hardness of Hadfield steels can be achieved through solid-solution heat treatment or plastic deformation. However, this increase in hardening can lead to the formation of crack cores and the future failure of the material. According to Hai et al. [3], when this material strain hardens by impact loading, the hardening mechanism is mainly attributed to the accumulation of dislocations and the formation of twins, without transformation from austenite to epsilon Martensite (HCP) or alfa Martensite, like in austenitic stainless steels [12,13].

De las Cuevas et al. [14], found that the effect of high temperature during heat treatments in high Mn steels generates a decarburization process on the surface, forming a considerable amount of Martensite and Austenite. During the decarburization process, the concentration of C and Mn decreases on the surface due to carbon diffusion, while the core retains the same initial carbon concentrations; the same effect occurs with manganese, but to a lesser extent. According to Wang et al. [15], in the decarburization process, the carbon atoms inside the material diffuse outward and react with the gases inside the furnace, inducing the loss of carbon atoms. It is important to highlight that alloying elements have a marked influence on the decarburization resistance of austenitic manganese steels, such as Mn, Cr, and Si. In this sense, Mn and Cr decreases the diffusion coefficient of carbon, thereby slowing down the decarburization process, whereas silicon has the opposite effect [16]. Therefore, the diffusion mechanism can lead to a heterogeneous microstructure made of Austenite and Martensite. Today, the studies of microstructure heterogeneity represent a new research field, where new hardening mechanisms are activated [17]. The objective of this paper is to study the effect of the protection efficiency against decarburization during heat treatment on the microstructural heterogeneity and the abrasive wear behavior of an ASTM A128 grade C austenitic manganese steel.

## 2. Materials and Methods

Austenitic manganese steel ingots melted in an electric furnace and sand casting were used for this study. Afterward, 14 specimens with dimensions 74 mm × 25 mm × 10 mm were machined according to the specimen size for wear tests based on ASTM G-65-16 standard [18]. The chemical composition was determined with X-Ray fluorescence using a Thermo Fisher Scientific Niton XL3T chemistry analyzer (Waltham, MA, USA) and optical emission spectrometry using an ARL ASSURE spectrometer (Waltham, MA, USA) (see Table 1).

**Figure 1:**
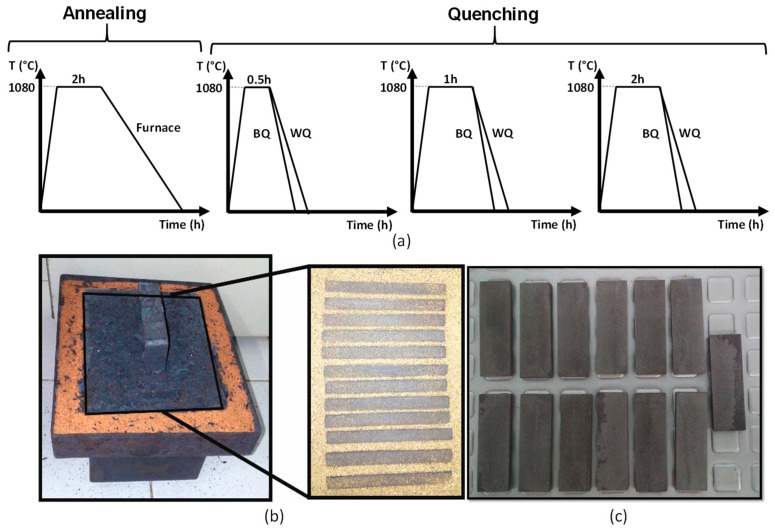
(**a**) Schematic diagram of the annealing and quenching heat treatment used in this study, (**b**) system of protection against decarburization with sand used in this study, and (**c**) specimens after thermal cycling.

In total, 13 specimens were subjected to homogenization annealing at 1080 °C for 2 h and cooled in a furnace for 18 h to room temperature. To prevent decarburization, the samples were placed inside a quenching box and were covered with silica sand. Afterward, 12 specimens were subjected to quenching treatment; 6 of these were protected in a quenching box covered with silica sand, and the remaining 6 were exposed to the muffle furnace environment. The austenitizing temperature used was 1080 °C, and the holding times evaluated were 0.5 h, 1 h, and 2 h. The quenching media used were 30% wt Brine (BQ) and Water (WQ) (see Figure 1 and Table 2).

For microstructural analysis, a Nikon MA100 optical microscope (Tokyo, Japan) and a HITACHI SU3500 scanning electron microscope (SEM) (Tokyo, Japan) were used. The analysis was performed on the cross-section of the specimens, which were previously prepared according to the guidelines of ASTM E3-11 [19], and were etched with dilute solution in water of HCl + HNO_3_ in 1:1 relation according to ASTM E407-07 [20]. For the chemical analysis of the second-phase precipitates, the Energy Dispersive Spectroscopy technique was used using an OXFORD Xmax probe (Abingdon, UK). As for mechanical properties, microhardness and abrasive wear resistance were evaluated. A STRUERS DuraScan 70 microhardness tester (Champigny sur Marne, France) was used; the load used was 500 g, and the indentation time was 15 s, according to ASTM E384-99 [21]. The analysis was performed from the edge to the center of the sample.

To analyze the resistance to abrasive wear, weight loss was evaluated by subjecting the material to the dry sand/rubber wheel test specified in ASTM G65-16 [18]. For this study, procedure E was used due to the hardness of the material. The operating conditions for this procedure were: 130 N applied load, 1000 revolutions wheel revolutions, and 718 m lineal abrasion. Finally, the worn surfaces were analyzed using scanning electron microscopy. The mass loss of the samples was measured on a Mettler Toledo ME204T analytical balance (Columbus, OH, USA) with a sensitivity of 0.0001 g. The values of volume loss were calculated according to Equation (1), with the density of the material as a given value (7.78 g/cm^3^) [22].

(1) $$Lost\;volume=\frac{Lost\;mass\left[g\right]}{Density\left[\frac{g}{{cm}^{3}}\right]}$$

The wear rate of the material was calculated according to Equation (2):

(2) $$Wear\;rate=\frac{Lost\;mass\;[g]}{Time\;[min]}$$

While the wear coefficient was determined according to the Archard Equation (3) [23]:

(3) $$K_{s}=\frac{3HV}{PL}$$

where: V: Volume of lost material [m^3^]; H: Hardness of material [Brinell]: P: Load [N]; and L: Sliding distance [m]. Because Equation (3) requires Brinell hardness, the hardness values were obtained by converting the values of the Vickers scale to the Brinell scale following the guidelines of ASTM E140-07 [24]. 

## 3. Results and Discussion

### 3.1. Microstructural Characterization

**Figure 2:**
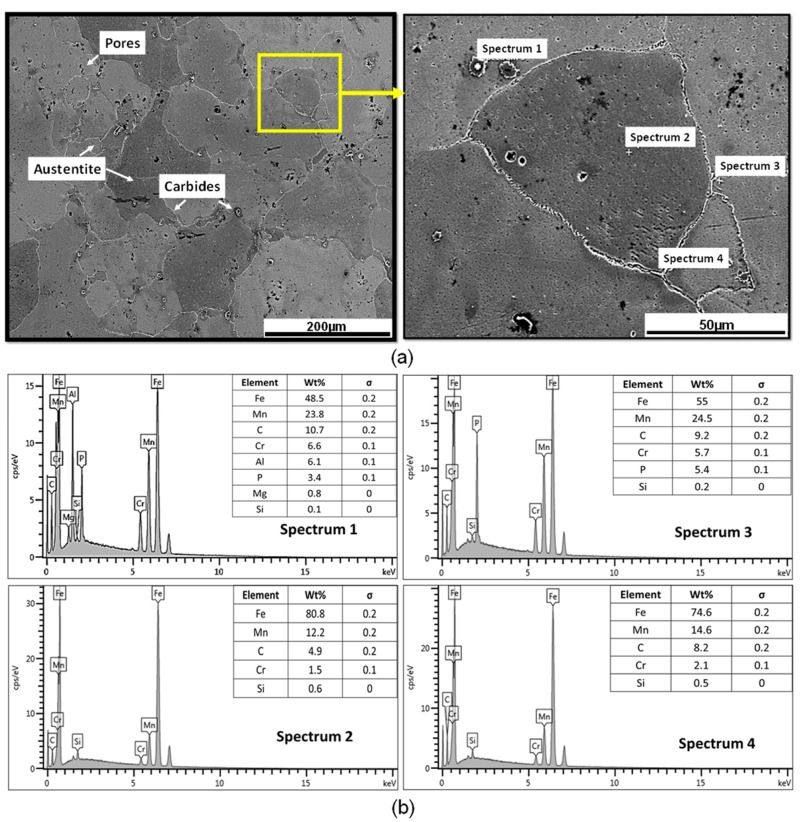
(**a**) Microstructural behavior and (**b**) EDS analysis of the austenite phase and carbides present in the grain boundaries of the material in the as-cast state.

Figure 2 shows the microstructural behavior of the material in the as-cast state, where the presence of a metastable austenitic phase with equiaxial grains of different sizes and very fine carbides precipitated at the grain boundaries is observed. The carbides present are of the type (Fe, Cr, Mn)_3_C and (Fe, Cr)_7_C_3_, considering the composition of the steel [23]. 

**Figure 3:**
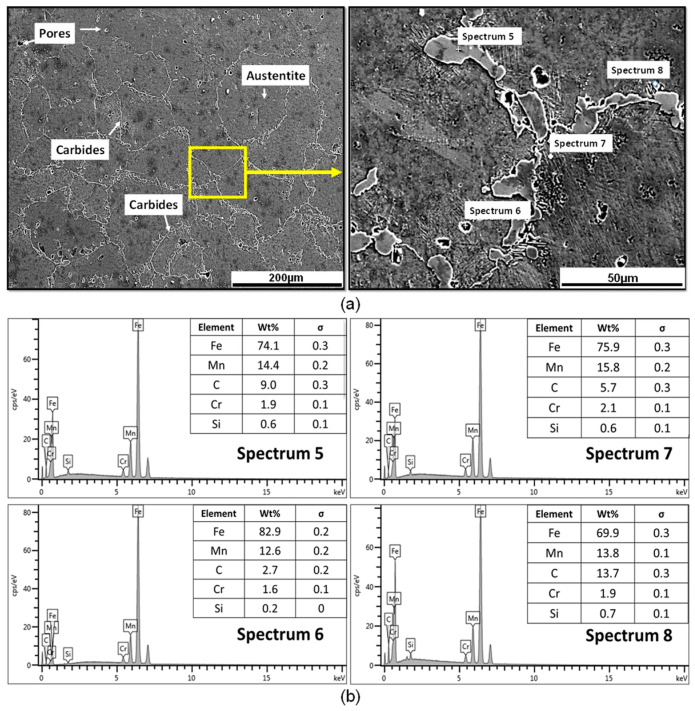
(**a**) Microstructural behavior and (**b**) EDS analysis of the carbides present in the grain boundaries of the material in the as-annealed state.

Figure 3 shows the specimen in the annealed state exhibiting equiaxial austenitic grains and a higher proportion of precipitated carbides at the grain boundaries compared to the material in the as-cast state. This is consistent with the thermal cycle applied. No evidence of any process induced by steel decarburization is observed. Analyzing the EDS spectra, the presence of complex carbides of the type (Fe, Mn)_3_C and (Fe, Mn)_7_C_3_ is noted due to the high weight percentage concentration of Mn and C reported. This behavior was documented by Torabi et al. [11], who reported (Fe, Mn)_3_C-type carbides in their EDS analysis with weight percentages of Mn = 15.07, Fe = 74.29, and C = 5.07. 

**Figure 4:**
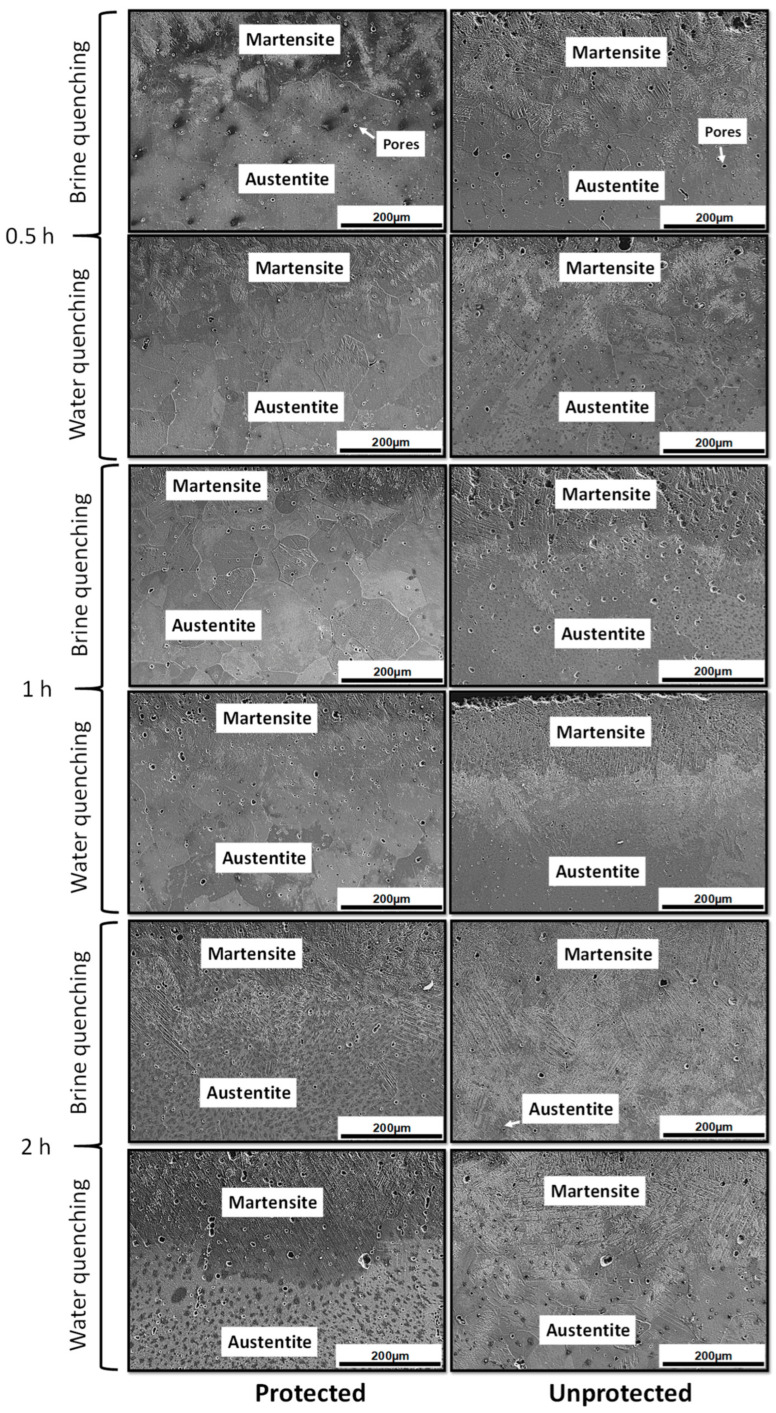
Microstructural behavior of the cross-section of specimens after heat treatment.

Figure 4 summarizes the microstructural behavior of water- and brine-quenched specimens after exposure at austenitizing temperature (1080 °C) for 0.5 h, 1 h, and 2 h with and without decarburization protection.

In all cases, the presence of Martensite induced by decarburization was observed, and this process produces a progressive decrease in carbon and manganese on the surface of the specimens, which enables martensitic solid-state transformations. The martensitic transformation was not greater due to the effect of chromium as a reinforcing element of the C-Mn pair, since the bond between manganese and carbon is not very strong and tends to break easily due to temperature changes. According to Higuera et al. [25,26], one way to reinforce such a bonding is to add an element that has a relatively high attractive force with carbon (chromium); therefore, chromium atoms become the bonding center of the weaker Mn-C pairs nearby, and form a much more stable austenitic structure. In addition to this, they reported that manganese atoms tend to occupy the center of the faces in the FCC cell. On the average, there is approximately one manganese atom in two FCC cells and one carbon atom in three FCC cells for these compositions. To ensure ordered Mn-C pairs due to fluctuations in alloy composition, it is suggested that there should be at least one carbon and manganese atom in the FCC cell, requiring a high carbon content in the alloys.

**Figure 5:**
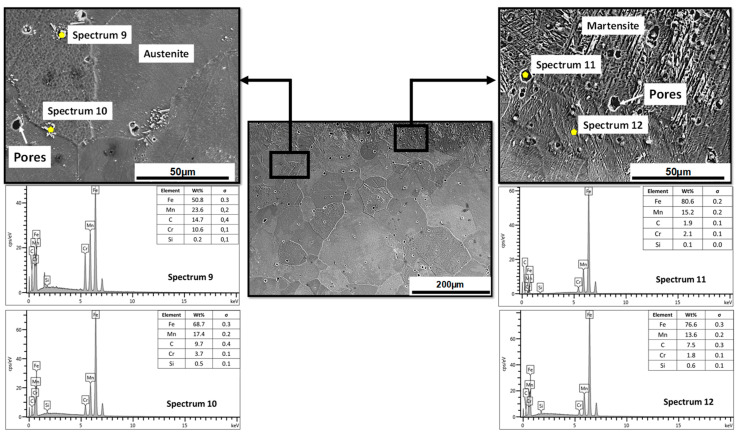
Microstructural behavior of the specimen in the protected condition with the best wear behavior after the material in the as-cast state. Specimen was austenitized for 1 h and quenched in brine.

Figure 5 shows the microstructural behavior of the specimen in the protected condition with a smaller wear rate (0.2355 g/min) and volumetric loss (151 mm^3^) after the material in the as-cast state. An essentially austenitic behavior is observed in the entire mass of the specimen. However, there is a 40 µm layer of Martensite induced by decarburization, which indicate that the sand protection used in this study was not completely efficient. When comparing the EDS spectrum number 2 of the material in the as-cast state with spectrum 12 of the specimen in the brine quenching condition, an increase of 1.4% in manganese, 2.6% in carbon, and 0.3% in chromium was observed, which could be an indication of the movement of the C-Mn pair in the direction of decarburization. The increased presence of Cr helped to prevent a disproportionate loss of carbon and manganese, and therefore prevented the formation of more Martensite. At depths greater than 40 µm, a completely austenitic behavior is observed with the presence, according to the EDS spectra, of some carbides of the (Fe, Mn)_3_C and (Fe, Mn)_7_C_3_ type. The formation of a heterogeneous microstructure across the sample thickness is supported by these observations. According to recent studies, heterostructured metallic materials are a novel category of materials that have the potential to disrupt established paradigms, including the incompatibility between strength and ductility [27,28].

**Figure 6:**
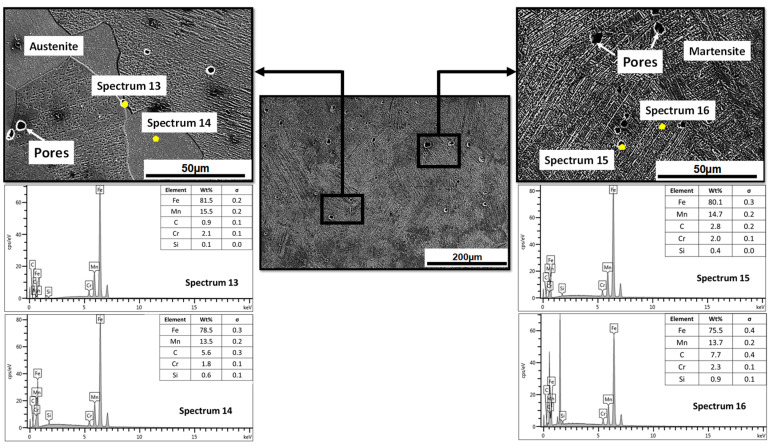
Microstructural behavior of the specimen in unprotected conditions with the worst wear behavior after the material in the as-cast state. Specimen was austenitized for 2 h and quenched in brine.

Figure 6 shows the microstructural behavior of the specimen with the highest wear rate (0.3202 g/min) and volumetric loss (206 mm^3^) studied in this work. The specimen was austenitized for 2 h in an unprotected condition and quenched in brine. It can be observed that the microstructure is almost entirely Martensite, with the presence of some islands of austenite supersaturated in carbon (5.6%) and manganese (13.5%) and others in the process of martensitic transformation. The martensitic layer observed exceeds 500 µm in some zones. This behavior was expected, due to the high temperature set in the austenitization (1080 °C) and the prolonged holding time (2 h) without protection against decarburization. The combination of these variables favors the decarburization and oxidation processes of the specimens, and therefore favors martensitic transformation. It is worth noting that a Martensite microstructure is not desirable in this type of material due to its high hardness and brittleness, which allows for a greater loss in volume during working conditions, thereby reducing the material’s wear resistance.

### 3.2. Mechanical Characterization

**Figure 7:**
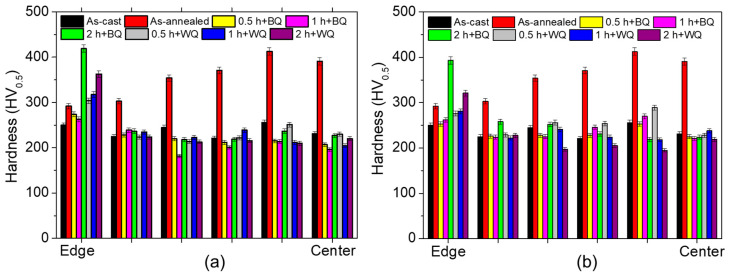
Hardness profiles of specimens in transversal section subjected to different austenitizing times and quenched in brine (BQ) and water (WQ). (**a**) Unprotected and (**b**) protected samples.

The first mechanical property evaluated was hardness, which is closely related to abrasive wear. The behavior of the Vickers microhardness from the center to the edge of the wear specimens was examined by analyzing the cross-section of each sample. The results are summarized in Figure 7, which shows an analysis of two groups of specimens, those protected and those not protected against decarburization, always comparing the material in the as-cast and the annealed conditions. The material in the as-cast condition showed the most homogeneous hardness values over the entire surface analyzed, while the annealed material in the protected condition increased its hardness towards the center of the piece; this behavior is consistent with the formation of carbides of (Fe, Cr, Mn)_3_C and (Fe, Cr)_7_C_3_, typical of a heat treatment that induces equilibrium in the material. This behavior is demonstrated in the paper by Maher et al. [7], where unconventional carbide structures with core–shell structures appear. It is observed that the inner part presents higher hardness (dark areas), and the outer part presents lower hardness (light areas), behavior similar to that observed in this research. On the other hand, Tecza and Solbule [6] carried out heat treatments of Hadfield steels at different temperatures (1100 °C, 1050 °C, and 1200 °C) showing that the formation carbide-free austenite is not achieved However, with prior annealing at high temperature, the grain size increases, which reduces its mechanical properties, but minimizes the carbide formation on the surface, before the subsequent treatment is applied. 

It is important to highlight that the material under study is an austenitic steel, and this phase is not characterized by its high hardness. As for the material subjected to thermal quenching cycles, a higher hardness was observed at the edge than in the center of the specimens, both in the protected and unprotected condition, which may indicate the presence of some transformations induced by the decarburization process that are undesirable in this type of materials. This behavior was more evident in the specimens that were held for 2 h at austenitizing temperatures (1080 °C), independent of the cooling medium used or their protection conditions. The formation of carbides of the type (Fe, Mn)_3_C present in the initial conditions of Hadfield steels leads to a reduction in mechanical properties such as impact strength and toughness [11]. However, by applying a heat treatment such as quenching, hardening in molten salts, high hardness at the edge (321 HV), and low hardness in the center (219 HV) of the specimens were obtained, generating a layer on the surface with good wear resistance. In addition, according to Bańkowski et al. [10], post-annealed treatments between 1000 and 1100 °C followed by rapid cooling allow the austenite to be released, but not to completely dissolve the carbides formed. The acicular form of these carbides may require extended treatment times to allow for the dissolution of the carbides in the austenite matrix. 

**Figure 8:**
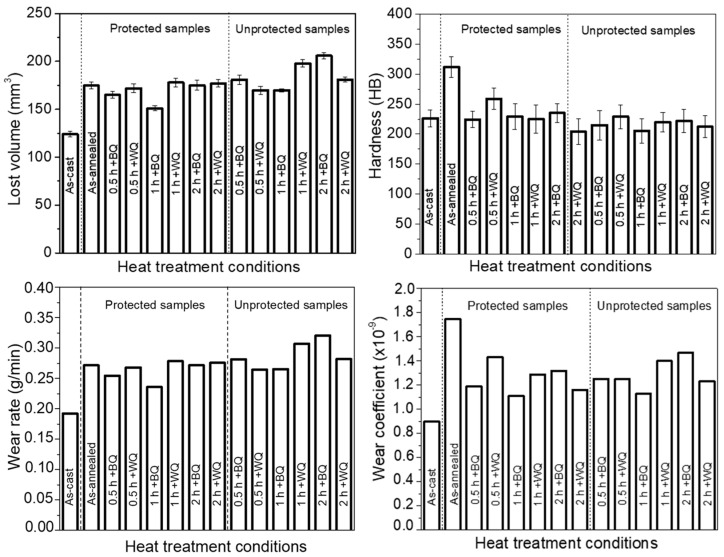
Wear behavior of austenitic manganese steel ASTM A128 grade C under study.

Regarding wear behavior, the results in Figure 8 show that neither of the thermal cycles studied improve the wear behavior of the steel in the as-cast condition with a wear rate of 0.1929 g/min, volume loss of 124 mm^3^, surface hardness of 226 HB, and wear coefficient of 0.90 × 10^−9^. The second specimen that obtained a good abrasive wear behavior was the one austenitized in a protected condition for 1 h and quenched in brine (BQ) with a 22.7% higher wear rate, compared to the material in the as-cast condition with a wear rate of 0.2355 g/min, volume loss of 151 mm^3^, surface hardness of 229 HB, and wear coefficient of 1.11 × 10^−9^. The third-placed specimen that obtained a relatively good abrasive wear behavior is the one austenitized in a protected condition for 0.5 h and quenched in brine (BQ) with 32.5% more wear rate than the material in the as-cast condition, with a wear rate of 0.257 g/min, volume loss of 165 mm^3^, surface hardness of 224 HB, and wear coefficient 1.18 × 10^−9^. As for the specimens not protected against decarburization, the specimen with the best performance was the one austenitized for 1 h and quenched in brine (BQ) with 38.1% higher wear rate compared to the material in the as-cast condition, with a wear rate of 0.2653 g/min, volume loss of 171 mm^3^, surface hardness of 205 HB, and wear coefficient 1.12 × 10^−9^. Quenching with water quenching allowed for a grain refinement of the austenite, compared to quenching in brine, thus achieving an improvement of its mechanical properties, as demonstrated by Mishra et al. [9]. In the case of this research, the best conditions were given with brine quenching and 1 h of heat treatment.

**Figure 9:**
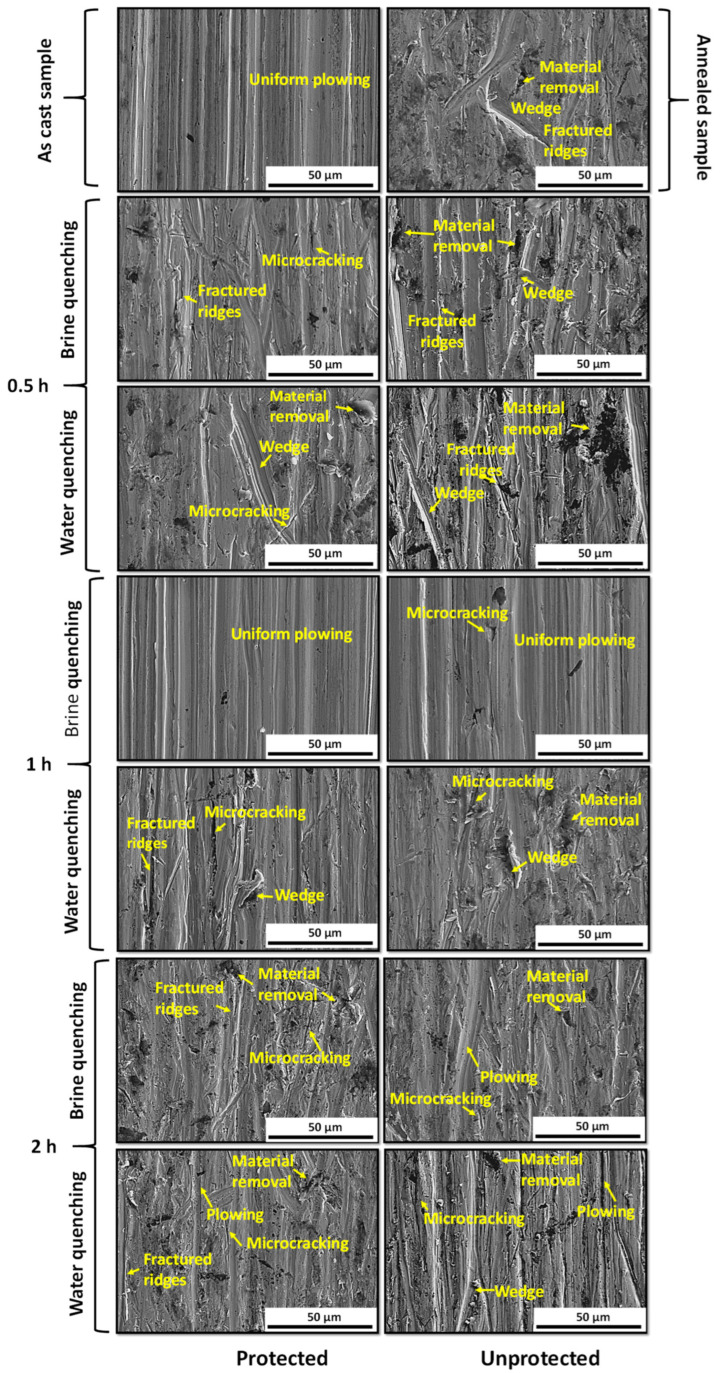
Micrograph of the worn samples’ surfaces generates under operation wear test parameters.

Figure 9 shows the behavior of the worn surfaces for the different thermal cycles under study. The results in Figure 8 are consistent with the results shown on the worn surface in Figure 9. The samples in the as-cast condition, and the austenitized one in the protected condition for 1 h and quenched in brine, showed good wear behavior with the presence of uniform plowing in all the mass of the specimen. In the rest of the samples, the presence of combined wear mechanisms such as micro cracks, fractured ridges, wedges, and material removal is noted. As for the material in the annealed state, the presence of several wear mechanisms is observed, evidencing the presence of second-phase compounds that brittle the microstructure, generating a greater loss of material with the presence of areas with fractured ridges and wedges due to its higher surface hardness (312 HB). The volumetric loss was 175 mm^3^, with a wear rate of 0.2716 g/min. The worst wear behavior was observed in the specimens that were austenitized without protection against decarburization and held for 2 h, independent of the quenching medium (see Figure 8). An increase of 66.8% in the wear rate was observed for the specimens austenitized for 2 h and quenched in brine compared to the material in the as-cast state with a wear rate of 0.3202 g/min, as well as an increase of 59.76% in the wear rate for the specimen austenitized for 1 h and quenched in water with a wear rate of 0.308 g/min. The wear mechanisms observed were material removal, presence of wedges, micro cracks, and plowing.

In conclusion, the results show that the hardness of the material is not a determinant factor in the abrasive wear behavior of the ASTM A128 grade C austenitic manganese steel under study, since the lowest hardness values showed the best wear behavior. According to Tressia and Sinatora [29], the predominant hardening mechanisms of Hadfield steels are stacking defects, twinning, martensitic transformations, and deformation bands, associated with their stacking failure energy, where the SFE of martensitic transformation should be around 18 mJ mol^−1^, and the twinning ones occur between 12 and 35 mJ mol^−1^. This twinning formation is associated with low stacking failure energies, explaining the work hardening effect of the steel.

## 4. Conclusions

Based on the microstructure, hardness, heat treatments, and abrasive wear evolution of an ASTM A128 grade C austenitic manganese steel, the following conclusions can be drawn:

1. The use of sand as a protection medium against the decarburization of an austenitic manganese steel during heat treatment was investigated. The results showed that the system used was not very efficient due to the presence of Martensite on the surfaces of the specimens. However, it is a good alternative if a controlled environment furnace is not available in the laboratory, because a significant reduction in martensitic layer thickness was found in the sand-protected material compared to the unprotected one, passing from layers of 40 µm to layers of more than 500 µm, respectively.
2. The effect of holding time at austenitizing temperature on the microstructural and mechanical behavior of austenitic manganese steel was investigated. The results showed that holding times longer than 1 h produced a significant increase of more than 100% in the thickness of the martensitic layer for both the specimens protected and unprotected against decarburization. This may be due to the oversaturation of carbon and manganese favored by diffusive processes from the center of the piece to the surface. The abrasion wear behavior of ASTM A128 Grade C steel was investigated. However, it is a good alternative if a controlled environment furnace is not available in the laboratory, because good microstructural and mechanical results were obtained in specimens quenched in brine after austenitizing for 0.5 h and 1 h. The wear rate values obtained were 32.5% and 22.7% higher than those of the material in as-cast state, respectively.
3. The effect of holding time on the hardness behavior from the center to the edge of the wear specimen was evaluated. The results showed that the hardness at the center of the wear specimen in both protected and unprotected specimens was uniform, with values around 220 HV_0.5_. While at the edge of the wear specimen, the hardness increased with holding time. However, a more significant increase was noted after 2 h of holding time, reaching surface values in the order of 343 HV_0.5_ and 419 HV_0.5_ in protected and unprotected specimens, respectively.
4. The abrasive wear behavior of an austenitic manganese steel after thermal cycling with and without decarburization protection was evaluated. The wear rate values obtained on the brine-hardened specimens after austenitizing them with protection against decarburization for 0.5 h, 1 h, and 2 h were 32.5%, 22.7%, and 40% higher than those of the material in the cast state, respectively. As for the unprotected material, the wear rate values were much higher than those of the material protected against decarburization in the order of 9.4% (0.5 h), 12.6% (1 h), and 17.5% (2 h).

## Figures and Tables

**Table 1:** Chemical analysis of ASTM A128 grade C steel under study, according to X-ray fluorescence, XRF, and optical emission spectrometry results.

Element	C	Mn	Cr	Mo	Ni	Si	P	Cu	Fe
Weight (%)	1.02	13.56	1.79	0.007	0.1	0.26	0.035	0.008	Bal.

**Table 2:** Heat treatment conditions.

Sample	Heat Treatment	Decarburization Protection	Austenitizing Time (h)	Cooling Media
1	As cast	--------	--------	--------
2	Homogenization annealing	Yes	2	Furnace
3	Quenching	Yes	0.5	Brine
4				Water
5			1	Brine
6				Water
7			2	Brine
8				Water
9	Quenching	No	0.5	Brine
10				Water
11			1	Brine
12				Water
13			2	Brine
14				Water

## Data Availability

The original contributions presented in the study are included in the article, further inquiries can be directed to the corresponding author.
